# Effects of Restrictive Bariatric Surgery on Congenital Prader-Willi Syndrome: A Case Report

**DOI:** 10.7759/cureus.27955

**Published:** 2022-08-12

**Authors:** Faiza H Soomro, Aneela Razzaq, Ghulam Siddiq

**Affiliations:** 1 General Surgery, The Dudley Group NHS Foundation Trust, Dudley, GBR; 2 General Surgery, Shifa International Hospital, Islamabad, PAK

**Keywords:** adolescents, bariatric surgery, prader-willi syndrome, hyperphagia, obesity

## Abstract

Hyperphagia leading to obesity is the most common cause of mortality and morbidity in Prader-Willi syndrome (PWS). It has been classified as the most common genetic cause of the development of life-threatening obesity resulting from a defect in satiety, with an onset during early childhood. Abnormalities in the feedback from gut peptides, including ghrelin, may contribute to the satiety defect; autonomic dysfunction may also play a role in impaired satiety. Usually, pharmacological treatment is ineffective in managing obesity in these patients. A 19-year-old male child with Prader-Willi syndrome presented with morbid obesity, obstructive sleep apnea, and impaired glycemic control. The patient had complained of hyperphagia since early childhood, but food intake increased aggressively in the last few years, which resulted in morbid obesity. The patient was treated with laparoscopic sleeve gastrectomy, and the residual stomach volume was 100 ml. The intervention resulted in a 37.1% weight reduction after one year of surgery with well-controlled blood sugar levels. The patient also reported improved overall quality of life, mood, and functionality. Laparoscopic sleeve gastrectomy can be offered to obese Prader-Willi syndrome patients with heightened mortality, particularly because no other effective alternative therapy is available.

## Introduction

The most common known genetic cause of obesity that is life-threatening is a multi-system congenital disease known as Prader-Willi syndrome (PWS) [1]. Almost one out of every 20,000 or 30,000 births has this disease [2]. It has a less familial tendency and is gender-neutral and sporadic [3]. A genetic anomaly causes Prader-Willi syndrome, which involves denovo deletion of chromosome 15q11-q13 paternally [4]. In about 35% of cases, maternal disomy fifteen or both chromosome 15s is seen. In the remaining individuals, this anomaly is caused by abnormal deletions or epimutations in the imprinting center of chromosome 15q11-q13 or some other 15s chromosome.

Severe infantile hypotonia, hormonal deficiencies like short stature and hypogonadism due to deficiency of growth hormone and other endocrine disorders, and obesity in early childhood that leads to developmental and behavioral anomalies if not responded to initially are primary symptoms of PWS. Other prevalent features include mild craniofacial abnormalities with dry mouth and enamel hypoplasia. Multiple autistic and behavioral characteristics, along with psychiatric phenotype, are also involved [5].

A multidisciplinary approach is used for its management, including a team of endocrinologists, geneticists, orthopedic surgeons, dietitians, primary care physicians, and mental health workers. They all work towards a collective goal of controlling obesity and other associated comorbid conditions. Hormonal replacement therapy and behavioral modifications are also included in its treatment. To correct hyperphagia and obesity, strategic vigorous diet plans and exercises are also included [6,7]. Rarely, bariatric surgery is needed when medical management fails to control weight gain. This facility is not widely available in Pakistan due to a lack of physicians' awareness, limited hospital resources, and financial burden on the family. Here, we present a unique case of morbid obesity in a patient with Prader-Willi syndrome that will end with successful weight reduction.

## Case presentation

**Figure 1:**
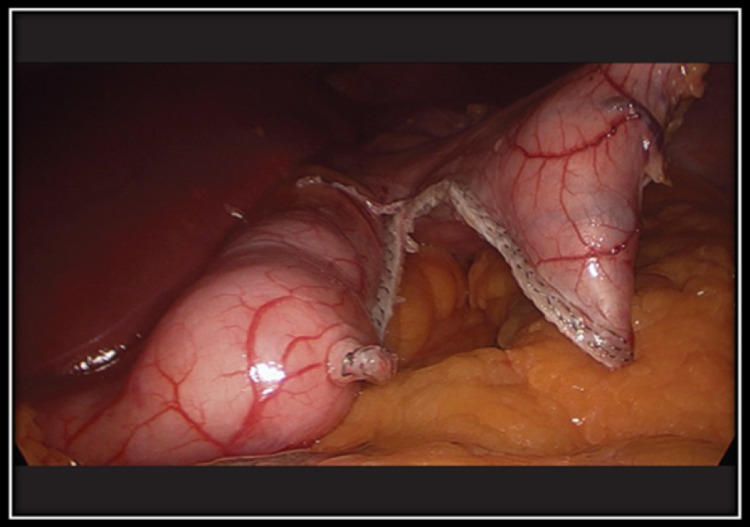
Demonstrating the residual stomach during the laparoscopic sleeve gastrectomy.

A 19-year-old male known case of Prader-Willi syndrome by genetic studies presented to our surgical clinic, referred by the pediatric team with complaints of progressive weight gain for the past two years. The patient was on follow-up in the pediatric medicine unit, where he was being managed for developmental delay and obesity. He was also under the care of a psychologist helping the patient with his eating disorder. The patient had complained of hyperphagia since early childhood, but food intake had increased aggressively over the past few years, which results in morbid obesity. Physical activity was decreased due to increased weight gain. A vicious cycle resulted in obesity and decreased physical activity. The patient had complained of feeling hungry all the time and kept on eating without any break. He admitted that he had difficulty following the diet plan and had cheated on hypercaloric meals and snacks. At the presentation time, the patient was morbidly obese with a weight of 156 kg, a height of 166 cm, and a BMI of 56.76 kg/m^2^. The patient was pre-diabetic with impaired blood sugar levels and increased blood pressure between the 95th and 99th centiles. The patient also suffered from obstructive sleep apnea and used a continuous positive airway pressure device (CPAP) at home. The patient also suffered from anxiety and labile mood. The patient was not responding to medical management, which included different diets and exercise routines, which eventually failed, so he was referred to our surgical clinic by the primary physician. Our surgical team assessed the patient, a pre-operative workup was done, and the anesthesia team was taken on board. Considering the clinical severity of obesity, his multiple comorbidities, and the failure of all previous weight loss plans, bariatric surgery appeared to be a reasonable and feasible strategy. Laparoscopic sleeve gastrectomy was planned under general anesthesia after discussing with parents for better patient management. The laparoscopic sleeve gastrectomy was done, and the residual stomach volume was 70 ml (Figure 1). Post-operative recovery was uneventful. The patient was kept on a liquid diet for two weeks following surgery.

On his first follow-up visit in the first week, he lost 9 kg. He didn't have any complaints. Over the next year, he had his regular follow-ups at one month, three months, six months, and one year with the surgical team and the psychiatrist. After one year, he has lost 58 kg and is off the CPAP device. Glycemic control was also facilitated. Percentage weight reduction was 37.1% in the first year after the operation. A comparison of presurgery and post-surgery characteristics with marked improvement has been shown in Table 1. The psychiatric analysis shows an improvement in mood and deterioration of anxiety and irritability. His speech becomes more enthusiastic about resuming daily activities. Overall, the quality of life was markedly improved, and the patient and his family were satisfied with the procedure.

**Table 1:** Demonstrating effects of laparoscopic sleeve gastrectomy on body mass index, CPAP usage, patient's mobility and QOL. QOL: quality of life and CPAP: continuous positive airway pressure.

	Pre-operative	Post-operative	Difference
Weight	156 kg	98 kg	58 kg
Height	166 cm	NA	NA
BMI	56.6	35.6	21
CPAP usage	Yes	Off CPAP	Resolution of sleep apnea
Mobility	Restricted due to joint pains	Started taking part in sports activities	Mobility significantly improved
QOL scale	04	09	Marked improvement

## Discussion

This reported case of a young patient with Prader-Willi syndrome is discussed that undergoes sleeve gastrectomy to treat severe obesity. It is still controversial whether adolescents should be treated with bariatric surgery or not, specifically in syndromic cases. Adequate data on the efficacy of other methods in treating obesity is still not available, but considerable evidence supports the effectiveness of surgical procedures. A multidisciplinary approach is directed towards treating syndromic patients, including a hospital bariatric surgeon and experts in psychological and physiological areas. The psychological evaluation is combined with the physiological one [8,9].

In this case, all the other methods of treating life-threatening obesity failed due to Prader-Willi syndrome. The patient was adamant about undergoing bariatric surgery, which was considered a last resort to improve his life. His parents were also supportive; they had all the limitations and barriers of Prader-Willi syndrome in their knowledge. A multidisciplinary team discussed all the possible complications, safety measures, and prognosis of this surgery. The uncertainty about future compliance and weight loss were also discussed.

The results from the 12 months of follow-up were encouraging. Different types of procedures are done as part of bariatric surgery in patients with syndromic obesity. Scheimann et al. in the meta-analysis reported that among individuals with PWS, 54% underwent biliopancreatic diversion (BPD), 29% gastric bypass, 5.4% jejunoileal bypass, 18% placement of a biocentric intragastric balloon, 3.6% vertical banded gastroplasty, 1.8% silicone band gastroplasty, and 1.8% truncal vagotomy with the division of the central nerve trunks slightly above the diaphragm [10]. Laparoscopic bariatric surgery is not very routinely done due to a lack of expertise and instruments.

In our opinion, laparoscopic sleeve gastrectomy is more effective and safer than other surgical procedures; it is associated with more significant weight reduction. Scheimann et al. [10] reported that weight loss occurs after different sleeve gastrectomy surgeries, like after using intragastric ballooning surgery, where a 14% decrease in weight has been said. Similarly, after gastric bypass surgery, a 4.2% reduction in weight was seen. Jejunoileal bypass resulted in a 19.3% weight reduction.

Patients undergoing jejunoileal bypass for weight reduction in PWS had increased adverse effects, including death, wound infection, and persistent fatty liver despite surgery [11-13].

In Martinelli et al. [14], similar results were reported as in our case. A 16-year-old male diagnosed with PWS was operated on with laparoscopic sleeve gastrectomy due to morbid obesity, obstructive sleep apnea, diabetes, and hypertension. After six months, weight reduction was 27.9%, improving glycemic control and blood pressure control.

## Conclusions

After laparoscopic sleeve gastrectomy, multiple children with Prader-Willi syndrome and adolescent undergoes weight loss along with the resolution of all comorbidities without any increase in mortalities. Therefore, laparoscopic sleeve gastrectomy can be offered to patients with this syndrome until there is a better alternative. Problems like osteoporosis, obstructive sleep apnea, and metabolic syndrome can be reversed by sleeve gastrectomy, which results in good quality of life, decreases complications, and increases life span in patients with Prader-Willi. Reduced body weight results in an improved body shape, enhancing self-confidence in these children.
